# An unclosed loop: Perspectives of community engagement in infectious disease clinical trials in sub-Saharan Africa

**DOI:** 10.1371/journal.pone.0308128

**Published:** 2024-08-02

**Authors:** Carmen Späth, Bey-Marrié Schmidt

**Affiliations:** 1 School of Public Health, University of the Western Cape, Cape Town, Western Cape, South Africa; 2 Division of Social and Behavioural Sciences, University of Cape Town, Cape Town, Western Cape, South Africa; 3 Health Systems Research Unit, South African Medical Research Council, Cape Town, Western Cape, South Africa; University of Oxford Nuffield Department of Clinical Medicine: University of Oxford Nuffield Department of Medicine, UNITED KINGDOM OF GREAT BRITAIN AND NORTHERN IRELAND

## Abstract

**Background:**

Community engagement (CE) is pertinent to ethically and scientifically rigorous infectious disease clinical trials in sub-Saharan Africa (SSA). However, there are critiques that CE is not properly embedded in research processes and that there is uncertainty about *what* CE entails. The aim of this study was to gain an understanding of CE in infectious disease clinical trials in SSA, specifically factors affecting CE and existing strategies for engaging with communities.

**Methods:**

Semi-structured telephone interviews were conducted with 20 community and clinical trial (CT) stakeholders who worked in SSA. The audio-recorded interviews were transcribed verbatim and analysed inductively using thematic analysis.

**Results:**

Themes are as follows: *1) Communities are abandoned research-entities*—a disconnect between scientific teams and communities was observed and knowledge translation was not prioritised at the community-level. *2*) *Us and them*: *community engagement teams vs investigators*—CE teams expressed that researchers did not account for CE processes and often did not involve CE staff in their planning, and felt that their roles were not valued. *3) Ethical considerations*: *concerns and gaps—*there were concerns that procedures were not standardised and that ethics processes were not adhered to. *4) Opportunities for improved CE practices*—training needs were expressed, including for standardised practices, ethics, and for developing a holistic understanding of collaborating with communities.

**Conclusion:**

CE role players require intensive training to ensure ethical CE and that communities are treated with dignity. This includes 1) using collaborative strategies involving research and CE staff, 2) protocol-adherence that recognises CE as pertinent, 3) viewing communities as complex and building relationships that are sustainable, and 4) ensuring that knowledge translation is considered at a community-level. Further research is necessary to investigate potential training programmes that integrate these elements.

## Introduction

Infectious disease clinical trials are essential to identifying treatment options for patients in sub-Saharan African contexts, where there are significant disease burdens related to HIV [1] and TB [2]. Clinical trial research cannot be implemented without research participants [3] and community engagement (CE) is the bridge between the research study and participants, and should be seen as an “ethical imperative” [4]. Community engagement is defined as an approach that aims to improve the conduct of research and its outcomes, through the ongoing collaboration of research-study staff and communities, throughout the research process (from protocol development stages to dissemination activities) [5]. These collaborations should be based on trustworthy relationships [6] and Dada and associates have proposed four R’s that should be embedded in CE processes, which (if implemented) are said to offer benefits for fostering effective engagement. Through the four R’s it is posed that *relationships* between CE trial staff and communities must be *reciprocal* and that communities should be approached in a contextually, culturally, and linguistically appropriate manner, and that *relatable* examples must be used to help people understand various facets of the study. Moreover, an appreciation of the “importance of interpersonal relationships and *respect* for the people, their customs, and traditions” must also be prioritised in CE processes [7].

CE practices have been positioned as essential to effective and ethical research and intervention processes [8]. Despite this, decades-long critiques persist problematizing the lack of integration of CE processes within research processes, including infectious disease clinical trials that have clinical side-effects for participants [9]. Earlier conversations by the research-community spoke to the need for literature and transparency about what CE entails in practice [9]. While this gap has been addressed to some extent, regardless of Good Participatory Practice, CE is still met with confusion stemming from unclear guidelines [10] and there is an evident need for transparency and clarification on the process.

A dominant narrative (and critique) surrounding CE is the need for ongoing (as opposed to once-off) relationships between clinical trial organisations/staff and communities, resulting in requests for building sustainable relationships among these parties [11]. CE stakeholders’ sentiments may hold undertones that poor “relationships” with communities threaten effective CE. For example, Dietrich and associates’ studies reveal important lessons as CE stakeholders repeatedly referred to the need for fostering connections, long-lasting relationships, and “finding the best way to reach people” that is not a “one-off” (2023).

Another aspect of CE that seems to be problematised is an apparent lack of consideration for communities’ identities (and the voices within these communities), and there is an expressed need for social, cultural, contextual, and geographical factors to inform CE [12]. CE should not operate without regard for the communities’ contextual, cultural, and social identity (among others), which can aid in facilitating effective engagement (12, 5). Communities themselves, as well as key representatives, i.e. Community Advisory Board (CAB) members who serve as a voice for the community and provide feedback on trial protocols, can help provide insight into these factors [13], and excluding these voices could threaten the trajectory and efficacy of the clinical trial research process. This was evident when CAB members deemed materials developed as part of a clinical trial recruitment process as culturally inappropriate and refused to share this with the community [5]. Consultation with communities/leaders/representatives throughout the study duration (5,13) as well as having an in-depth understanding of communities and their needs [7] should not be considered as secondary parts of CE. Instead, engaging with these parties can promote effective CE through these individuals advocating for health research studies [6], which might have implications for participation in clinical trials. Considering this, formative research into the socio-cultural and political factors that are characteristic of a given community can help cultivate a holistic understanding of communities as unique systems (4, 12). Knowledge of community-level factors that hinder or facilitate engagement can inform approaches to building sustainable connections with communities which can benefit clinical trial procedures [4].

While there is still work to be done in conceptualising and adapting CE processes appropriately within clinical trials, it should be acknowledged that strides have been made to clarify current CE practices and providing context-specific guidelines for this [13]. However, Good Participatory Practice (GPP) appears to require further development to account for CE stakeholders who are not clinically-trained investigators, but who rely on these guidelines (i.e. fieldworkers or community liaison officers). The latter refers specifically to the current GPP guidelines for TB and HIV biomedical research, which are markedly presented in a theoretically and scientifically-focused format that is not necessarily suitable for all staff and community representatives in this research [14–16].

The need for these guidelines is tied to advocation that CE must be considered as central to effective biomedical research processes that may have implications for infectious-disease treatments [6]. This seems to be crucial in SSA contexts with a high burden of diseases like HIV [1, 17]) and TB [2]. Moreover, it has also been highlighted that CE may not only strengthen possible engagements in other trials, but that building relationships and trust with communities may also have positive implications for how treatment options are perceived for disease management outside of the trial (i.e. vaccine uptake [3]).

Further elucidating the centrality of CE processes within infectious disease biomedical studies, Simwinga and colleagues (2016) distinguished between two key approaches and associated activities that can facilitate both effective and ethical research. This includes 1) *programme-wide activities* (educating communities about the research and their rights), 2) *programme-specific activities* (geared to the requirements of the study), 3) *promoting intrinsic activities* (building respect and trust with participants and involving key stakeholders), and 4) *instrumental activities* (strengthening the quality of the research through sustaining interest and improving the consent process). While these concepts, GPP guidelines and existing literature helps the implementation of CE within clinical trials, there are still important gaps that need to be filled on this topic, including the lack of reporting and evidence on how CE processes are implemented [4].

A need remains for understanding CE from the perspectives of those who are involved with it, to foster an insider-informed understanding of these processes, which may contribute to strategies that can enhance CE in clinical trials. For the current study, we posit that to understand how CE activities unfold in practice (or do not), it is necessary to learn from the experiences of CE stakeholders, including research-staff, CE staff, CAB members, research participants and community representatives. The aim of this study was therefore to gain an understanding of CE in infectious disease clinical trials in SSA from the perspectives of key stakeholders, focusing on factors affecting CE processes, and existing engagement strategies.

## Study methods

We conducted a qualitative research study, which entailed semi-structured telephone interviews with 20 CE stakeholders involved in infectious disease clinical trial settings across SSA.

### Participants and recruitment

Participants for the study were purposively selected to ensure that various CE stakeholders’ perspectives and experiences were represented. In terms of the inclusion criteria, participants needed to work/have worked with clinical trial studies in SSA or have been a participant in a clinical trial in SSA. Examples of the types of participants earmarked for inclusion included community liaison officers, fieldworkers, researchers, clinicians/health care workers, community representatives, CAB members, trial participants, community engagement directors/managers, and clinic/site managers. Global guidelines for good participatory practice of CE refer to those “who have a stake in a biomedical HIV prevention trial” as stakeholders [15]. We agree with this term, but also note that CE teams are stakeholders whose primary responsibility is CE including those who manage CE teams (directors/managers), those who coordinate trial activities (coordinators) and those directly engaging/interacting with communities (liaison officers/fieldworkers/CE workers). Other CE stakeholders are staff who are investigators, clinicians, academics and CT operation managers. Furthermore, other stakeholders include trial participants themselves and individuals referred to as CAB’s. These individuals volunteer to serve as an “independent advisory voice and facilitate community stakeholder participation and involvement in the research process” [15].

The researchers invited potential participants (all who were above 18 years of age) via the following avenues: the South African National Clinical Trials Register (SANTRC); the Pan African Clinical Trials Registry (PACTR); the Vaccine Advocacy Resource Group (VARG); the International AIDS Vaccine Initiative (IAVI); and social media (Twitter/Facebook). In total, 20 different organisations/councils/research institutes were approached for participant recruitment. The following methods were used for contacting these parties: 1) organisation representatives/stakeholders/collaborators were contacted and provided with a comprehensive overview of the study, 2) they were asked whether they could disseminate study information to their colleagues/networks and provided with this information (including flyers, information sheets and consent forms which were prepared in English, isiXhosa and Afrikaans), 3) they disseminated this information as well as a Google Form whereby potential participants could express their willingness to participate and indicate consent for the researchers to contact them. Following this process, the researchers reviewed the Google Form to which 190 people responded. We contacted all interested individuals via email, invited them to participate in the study, and emphasised that their participation would teach us valuable lessons for strengthening community engagement in infectious disease clinical trials. Of the people who indicated their interest in participating, less than half responded to the email. After negotiating times with interested people, we were able to interview our envisioned sample of 20 CE stakeholders. The recruitment phase for this study commenced on 1 February 2022 and ended 30 September 2022.

### Data collection

This is an explorative qualitative study, with both researchers having experience in conducting/teaching qualitative research methods. During data collection, both researchers conducted individual interviews via Zoom or telephone calls and all participants were comfortable with speaking English. These semi-structured interviews were audio-recorded if permitted by the research participant. The researchers used a set of questions outlined in an interview guide and used probes throughout the interview, which helped them to learn more about the topic of community engagement from various stakeholders. The interviews varied in length but were on average 60 minutes each.

The questions captured in the semi-structured interview guide contributed to understanding various facets of CE in clinical trial research and the researchers were able to gather meaningful and valuable data about the barriers, facilitators, and strategies of community engagement at various clinical trial sites in SSA. Questions included, ‘Can you please tell me about an activity or process that you were involved in that led to effective or successful community engagement?’ and ‘What are examples of things that make engagements between clinical trial and community stakeholders difficult or challenging?’.

### Data analysis

The 20 audio-recorded interviews were transcribed verbatim by a professional transcriber who signed a confidentially agreement to protect the rights of participants. The semi-structured interviews (conducted in English), were analysed using thematic analysis, which was largely informed by Braun & Clarke’s (2006) approach to analysing qualitative data. Thematic analysis enables qualitative researchers to identify, analyse and report the patterns that emerge from interview-data [18]. This process therefore aided the researchers in sharing the perspectives of people in terms of their experiences and perspectives of CE in clinical trial research.

Using the principles of thematic analysis, the first author familiarised herself with the data through active reading, which helped her get a sense of the breadth of the data. Thereafter codes were generated manually (using a notebook, pens, and sticky notes) and digitally through using the qualitative data analysis software, Atlas.ti. Notably, both inductive and deductive coding were used to ensure that the data speaks to the research question, but that there was equal room for codes to emerge organically. Codes were systematically considered, sorted, and combined to form themes that identified meaning within the data. Themes were then reviewed, named, and defined and the author ensured that they reflected participants’ narratives.

To add to rigour to the data analysis, the aforementioned process was preceded by a preliminary coding phase. During this process the first author analysed a few transcripts using thematic analysis and created a code-book. These findings were shared with the second author, who provided reflective feedback. A research intern also contributed to this process by reviewing the codes generated for the study as well as the findings. The intern agreed with us on all aspects of our analysis and provided rich ideas for writing this paper. It is noteworthy that while this code-book informed the final data analysis process, codes were not strictly assigned to this structure.

### Ethics

Ethics approval was granted by the University of the Western Cape’s Biomedical Research Ethics Committee (BMREC). In conducting this study, we adhered to the guidelines provided by the POPI Act (Protection of Personal Information Act, 2019), the South African Department of Health’s (DOH) ethical guidelines for health research [19] and the Helsinki Declaration [20]. Each participant provided written consent prior to participating in the interviews.

The researchers aimed to protect the identities and nature of participants’ contributions. Confidentiality and anonymity were ensured through the following methods: participants’ names were not included in the reporting of results; digital copies of data were stored on password protected computers and online data-storage platforms; files were password protected/identifying information was removed; hard copies of data were stored in a locked cabinet.

Participants were informed of their rights and that participation was completely voluntary. They were assured that they could stop participating at any time without consequences and were given the contact details for the ethics committee. In addition, they were informed that, should they experience distress while sharing their experiences, that appropriate referral to a mental health care worker would be made.

## Findings

### Demographic characteristics

Participants are described in Table 1. We conducted 20 in-depth interviews with participants who worked in SSA, particularly Nigeria, South Africa, Uganda, Rwanda and Ethiopia (notably, some individuals worked across more than one setting in Africa). Participants included staff primarily responsible for CE, including CE managers, CE coordinators for trial activities, CE workers (e.g. responsible for liaison with communities) and some heads of CE. Participants further included investigators, including researchers, clinicians/academic staff and a head of clinical trial operations.

**Table 1:** Participant characteristics.

Participant number	Role in clinical trials	Organisation/ institution	Age	Declared gender	Sub-Saharan African country
1.	Community engagement (CE) manager	Research institute	50	Female	South Africa
2.	Clinician and academic	Clinical trial Registry	37	Male	Nigeria
3.	Clinician and academic	University teaching hospital	Not disclosed	Not disclosed	Nigeria
4.	CE manager	Implementation projects	38	Male	South Africa
5.	Head of CE	Research institute	45	Male	South Africa
6.	CE worker	Research institute	Not disclosed	Not disclosed	Various—SSA
7.	CE manager	University research division	31	Female	South Africa
8.	Head of CE	Research institute	42	Female	Uganda
9.	Researcher	University research division	Not disclosed	Not disclosed	South Africa
10.	CE manager	University	42	Male	Ethiopia
11.	Researcher	Research institute and University	43	Male	Rwanda
12.	Clinician	Research institute	Not disclosed	Female	Nigeria
13.	CE manager	Research institute	55	Female	South Africa
14.	Researcher	Research institute	47	Female	South Africa
15.	Clinician	University	57	Male	Nigeria
16.	Researcher	University	45	Female	South Africa
17.	Head of CE and research	Infectious disease vaccine network	57	Female	Based in United States, works across SSA
18.	CE coordinator (communications, marketing and CAB coordination)	Infectious disease vaccine network	54	Male	South Africa
19.	CE coordinator (health forum coordinator)	Health forum	23	Female	South Africa
20.	Head of clinical trial operations	Clinical trial organisation	47	Male	Various—SSA

### Findings

The perspectives of CE stakeholders revealed four themes, labelled as follows: 1) Communities as abandoned research-entities, 2) Us and them: community engagement teams vs investigators, 3) Ethical considerations: concerns and gaps, and 4) Opportunities for improved community engagement practices. These findings, which emerged via a thematic analysis, are presented below.

#### Theme 1: Communities as abandoned research-entities

The key aspects related to theme 1 are that CE teams (e.g. liaison officers/fieldworkers) believed that communities (e.g. study participants) were often abandoned by clinical trial researchers following enrolment. This is tied to the critical sentiments that some researchers only saw participants as *numbers* for their studies.

A recurrent theme among respondents was an apparent disconnect between community members (e.g. potential participants) and researchers. One respondent explained that this disconnect occurred because research teams: “…do research *in* the community, they are not doing research *for* the community”, describing that CE was viewed as a means to an end (enrolment) and that participation in studies serve no benefit for participants or the broader community.

Echoing these views, another respondent referred to this disconnect as an “abandonment” or an “unclosed loop” where researchers viewed their relationship with trial participants as only valuable at the initial stages of a trial, but not towards the end of a trial (e.g. when reporting the results or about the next steps following study-closure).

*The other thing that I find is that once you [CT staff] are there, they always feel that you come there when you want something, but you don’t come when it’s done and … so for me community engagement is sort of, I would imagine, closing a loop, and we never close the loop.* [Researcher]

Respondents expressed that as part of CE, clinical trial teams sometimes viewed community members as merely “numbers” for studies. Moreover, one person likened clinical trial teams to “vampires”, as collecting blood samples seemed to be their only priority and not the people themselves. This respondent held a senior management role in CE at an infectious disease institute:

*We come for bloods in community level…so to change this vampire mentality then we need to work with the com[community]… if you are a doctor and you do not have the community at heart, you are just a villain.* [Head of CE]

Another respondent in a similar managerial post in CE described how current research practices do not translate to sustained communication with trial participants. They noted that when CE is initiated at the start of a study, clinical trial staff are said to be “vibrant”—seemingly excited about engaging with the community. However, after the required sample has been enrolled, CT staff no longer show an interest in the broader community and are said to “go silent…until there is another study”.

*For example, you have enrolled your 45 people that you needed for your phase one, then you get silent. You only concentrate on the 45 people that you recruited and you don’t mind about the immediate communities where you recruited these people from in terms of giving them feedback.* [Head of CE]

In agreement with the concerns discussed, various respondents suggested that the level of knowledge dissemination at the community-level was insufficient. An investigator working with infectious diseases described that there was sometimes a level of disrespect for communities since community members/participants were not informed of outcomes related to the study sufficiently, whereas other stakeholders (such as researchers) had access to academic outputs for the study. The respondent was angry about how CT staff treated participants and used repetitive words to express her dissatisfaction: “…not once, *not once* did they say thank you to all of the people that participated”.

*Respondent: we don’t go back to close the loop?*

interviewer: why do [you] think it is…

*Respondent*: *I think because dissemination for a researcher is publication*, *right*? *And*, *but we must also be aware of the fact that publication only reaches … so for us that is closing the loop*, *but it’s actually not because at the end of the day*, *what I’m referring to*, *is letting the people know*. *You know*, *the people that we started off with*, *letting the people know this is what happened…If I think about subsequently to rolling out when they were doing [the roll out of a certain programme]*, *we had the interviews [on the news…we had the this*, *we had the that*, *we had the head of you know*, *the people in politics saying ah*, *you know*, *we’re rolling out [this programme] but in any of those things*, *not once*, *not once did they say thank you to all of the people that participated*. *We say thank you at the beginning*, *we say thank you when we’re following up*, *we say…but at the end we don’t say*, *and you know*, *those people that participated in that trial thank you very much*. [Researcher]

Conversations about the community being left behind was also evident when a respondent spoke about CT staff dishonouring their agreement to share health information with community members, as promised during initial CE activities. Their perspectives touch on the *silence* towards community members mentioned previously:

*People have questions to ask–they are not seeing you (the CT staff) anymore. Yet, initially you built a relationship where you mentioned to them that you will be able to provide them health education, you will be available to answer their health concerns.* [Head of CE]

Related to the latter issue of accessible information, a respondent underscored the need for knowledge translation that is appropriate for “ordinary South Africans”–alluding to the importance of sharing scientific information with participants and the community in an accessible format. They noted that having access to health-related educational resources and sessions after the trial would have been important for the welfare of communities, but that there was a gap in achieving this outcome.

*But also, part of that translation I was talking about, is the issue of how do these studies start to make sense for ordinary South Africans. One, are we making these studies just because we want money as scientists or, because they, anyway, they come as a grant isn’t it? Or either are we making these studies because really, we want to effect or affect the HIV status quo and change the route of HIV? And how does it make sense then? How then do we start to marry the two? And also how then do we make, bring in the voice of scientists and doctors and everyone who is researching at the community level? And then into community also to start to chat, to speak with the community and let the community have the first-hand information and let the community ask them at first hand.* [Head of CE]

Tied to the concerns that communities are not adequately informed about the important study-outcomes, some respondents described their role in this matter. In particular, a CE manager noted that their role was rooted in being a bridge towards tangible and beneficial outcomes for communities who are associated with clinical trials. A head of CE expressed similar notions, describing themselves as a role player and having a “duty” to make room for community collaboration.

*[My] duty then is also to bring them along into these spaces. I create spaces when there are no spaces, I build platforms where there are no platforms. Then just to make sure that science is not just for scientists, but science is for the community. But also then looking at ways and means in which [one] can utilise them in building the community voice through investment.* [Head of CE]

On the matter of discussing responsibilities, it was apparent that CE teams who were primarily responsible for liaison, fieldwork, and CE work were critical about how researchers perceived staff who were responsible for CE. These respondents alluded to a disconnect between these parties, which is discussed in the next theme.

#### Theme 2: Us and them: Community engagement teams vs investigators

Theme 2 specifically relates to the views of community engagement teams (e.g. head of CE; CE manager). Notably, a dominant narrative across the interviews was that there seems to be a disconnect between community engagement teams and investigators (researchers and clinicians). This disconnect was underscored as problematic by CE team members, who felt that their role and involvement in clinical trials were not valued by researchers, partially due to apparent failure to involve CE teams at an appropriate and early stage of a clinical trial. Moreover, in relation to this, one head of community engagement expressed disappointment that CE teams invest a lot of time and effort in engaging with communities and potential participants, but that this is overlooked once research teams have enrolled participants.

*Community engagement teams get into the field, make all the efforts that they do, they trail all around the different stakeholders and when they start these trials the researcher are able to recruit for example 40 people in maybe 3 months and they clap their hands and that is success for them because they have been able to enrol and have their people on, but the community engagement team have actually succeeded because they made this happen but because they don’t document what they do and how they did it and how it lead to this success, their worked is look, is given little attention. So I think what we need to also do is put a lot, some effort around supporting community engagement teams to appreciate the value of document community engagement, activities that they do.* [Head of CE]

For some this was evident through their overt exclusion from key research processes including those related to decision making about CE procedures. A CE manager at a research institute also seemed to imply that CE was treated as an ‘after-thought’ in the research process. For instance, it appeared that the investigators delivered a presentation about the clinical trial to the CE team after they had already started enrolling participants, as evident below.

*No study should be implemented without presenting to the CWG [refers to the community working group who are “responsible for ensuring that the principles of community involvement are the foundation of all community engagement activities at each site and facilitating community participation throughout the research process”* [21]. *Sometimes…there is a scientist who will say, oh, okay, thanks for having me here, I’m going to present a study, and there’s no PowerPoint…the scientist is just talking. And then she will say, “oh, I’m sorry, I need…to quickly go back, we are busy with the enrolment for the…for the same study that I’m presenting, okay, so and so will proceed”. How do we do an enrolment while you are coming to do the first presentation to the CWG members? Yes, so that’s one of the challenge[s].* [CE manager]

Others noted that it is because of research team members’ perceived view of CE as restricted to recruitment that CE teams felt their roles at work were often viewed as limited and as an ‘advertisement’ process that could affect how the role of CE teams are valued by other CT staff. A CE manager/supervisor expressed that:

*I think the people really appreciate services being brought to them because in the clinic you know, first of all to get to the clinic is, you have to pay transport, the weather is not always playing with and it’s overcrowded, there is not necessarily space to sit. So just the convenience and then also just the fact that there is an activity happening, the kids are always very excited to see you know, we are there, there is music, we give them lollipops or… and then for the adults you know, there is the engagement and it’s really valuable having community members that we already trained, who are capacitated to talk about not necessarily research. I never go out necessarily, not never, I’ll never say never, but the goal to go out is not to advertise a specific study. It’s more to build a relationship with the people. It’s about TB and HIV, so our goal is to, you know, raise awareness and encourage the testing.* [CE manager]

*So*, *I feel like sometimes [departments]or a sponsor are very much disjointed*, *so very siloed*. *So then the protocol writing team wouldn’t always know to involve us*. *So that gap in communication is frustrating sometimes and the way that we are addressing that is to write our processes into the SOP’s*, *so then it becomes a mandatory thing to involve the community engagement contact in some of the other communications to make sure that we are part of the initial discussions and not only find out about it later*. [CE worker]

Narratives of exclusion were also evident when CE team members critiqued how communities are approached by ‘other’ CT staff. Some CE team members discussed how strategies for appropriate and effective engagement with communities were not prioritised in research-planning, which was said to manifest due to investigators failing to view CE as an essential aspect of research.

In relation to how communities should be approached by researchers, some respondents drew attention to an apparent lack of respect for community members, their complexities, lives, and environments. Through the conversations it appeared that the “us vs them” theme seemed to emerge particularly since CE staff aligned themselves with the community members instead of with the CT staff they are working with, as suggested below.

*My duty also is to link science and the community, because when,…my first criticism of the researchers and scientists is that they always leave the community behind and sometimes they speak gibblish, things that we don’t understand. So it is then my duty then to start to then to translate that with what does it mean in the ordinary language for ordinary people.* [Head of community engagement]

This point introduces the next finding (theme 3). This section explicates critiques of current CE procedures and introduces ideas for addressing ethical issues related to research teams and all CE stakeholders.

#### Theme 3: Ethical considerations: Concerns and gaps

Theme 1 (communities as abandoned research-entities) already captured one ethical concern raised by some respondents–that communities appear to be abandoned after recruitment, enrolment, and data collection has been completed. Notably, multiple respondents made remarks about the centrality of ethical principles and processes for themselves, and critiqued existing ethical guidelines (and the lack thereof) within their workplaces. A CE team member whose role is solely focused on community development and health-empowerment (and no research processes) shared the following views.

*This is the problem with the way we run these research trials—we firstly never understand, especially in the sort of under resourced communities, communities that depend a lot on the social system. And they [community members] know that our research has got findings and implications for that system, they know that the public health system uses the data that we collect from them. And if we do a show of how we engage policy makers or even local level decision makers on the findings, they don’t really buy into what we are doing.* [CE worker]

Elucidating this finding, a respondent illustrated the importance of ethical guidelines by providing some scenarios they have faced, and explaining how contingency measures for referral were critical (i.e. law enforcement and psychologists). This person discussed how concerns with the stigmatisation of study participants by community members, along with a concerning number of domestic violence cases, were prominent among their study participants.

*So, we had incidents where participants were stigmatised. So for instance, they would go to the research site that used to do, for instance, only HIV treatment trials, and when they come in for a TB vaccine, and then the participants are shunned and said they have HIV and then we just have to support those kind of participants with that. We have also heard of participants where the study staff become aware of like, for instance, domestic violence or other issues–I mean there are so many issues in the communities that we work with–where sites also have to deal with that. And so then we also make sure that, in the beginning, that the referral systems are definitely in place so they also know how to deal with these situations.* [CE worker]

Respondents also expanded on ethical concerns pertaining to CT participants themselves, with one individual alluding to concerns about participants not sufficiently understanding the research concepts discussed, raising issues about whether it is then appropriate to accept their informed consent. Related to this, another implicit example of potential insufficient/ inadequate information being shared was provided from a CE manager who stressed that research processes involving potential participants must foster their knowledge of the research study, its objectives, procedures, and implications.

*We made a video where we filmed the whole process, explain how it works, the risks involved with that, so participants can really know what they can expect of it, before they sign up for the trial. Where they aren’t shocked by something that needs to happen. And I mean Mucoses sampling and fine needle aspiration and use of the soft cup in teenage girls where we want to collect mucoses samples and that’s all really controversial, invasive procedures. So to make sure that people are really fine with it and that people know what’s going to happen and that the community also understand why it needs to happen.* [CE worker]

In other instances, it appeared that gaps in protocol adherence stemmed from inexperience or lack of knowledge of some research concepts, among staff, raising concerns about whether they should be communicating with participants while having these knowledge gaps. In this case, one participant, who described themselves as “well-educated”, problematised the expectation of staff to provide feedback on research matters when they do not understand what they term as “research language”, including concepts like “randomisation, informed consent and placebo”, which they themselves (as a CE manager) found challenging.

*Ultimately, I also remember when I started in research as a person who is, you know, was qualified, a professional, well-educated, and it was so difficult for me, you know, research has their own language. If you’re in here you understand it, but just think back that, you know, in the beginning it can be overwhelming and we’re taking about studies and it’s numbers and all these randomisation, informed consent and placebo and it’s a lot of…and were expecting people, for instance, to give feedback on protocols or to partner with us and, you know, we can’t expect them to do that if we don’t fully understand, if they don’t fully understand, you know, what the purpose is, what we’re doing, what we’re talking about.* [CE manager]

Respondents outlined how work at the sites are “siloed” and “disjointed”, with some CE team members noting their exclusion from important planning processes irrespective of the standard operating procedures specifying the compulsory involvement of CE teams. Moreover, a CE worker particularly mentioned their exclusion from protocol-writing and expressed concern that they are expected to develop presentation slideshows to explain the different components of the study to CAB members without having been involved in early and essential stages of the research process. This draws into question whether there are any ethical considerations attached to secondary involvement of CE teams, who are a direct link to community stakeholders like CAB members and potential participants.

*The protocol writing process is such a pressured time, so to make sure that there is like, can be a CAB meeting at every site in time takes a while but also it is a really hard thing to juggle. And what we do for the CAB meetings in the beginning, so we, during, while the protocol is being written, we like nowadays we are involved–they do involve us as part of the protocol writing team and then we develop slides to explain the study. So it’s very, it’s the protocol simplified and it covers all the topics that CABs usually want to look at. So it’s not only the information that’s in the protocol–that’s other topics as well and then we address as many of those in the slides already and then the sites can use that to present to the CAB and then gather questions on specific topics and then we track those and make sure that they all are addressed.* [CE worker]

Further implied ethical concerns regarding research team members emerged through expressed needs for training in administering informed consent procedures amongst fieldworkers, while a research clinician similarly described a crucial need for pre-study training among clinical team members, noting that this will “sort of create(s) a levelling ground for all clinical trial personnel, all the personnel working on a clinical trial”. What is evident is a recognised gap in knowledge, or rather opportunity for further development for staff engaging in all research procedures, reflected in the clinician’s view below.

*Well, potentially we, prior to administering informed consent, there would be a presentation through the service and a pre-study training for would-be assessors as well as people who would need to retrieve, who would need to collect consents, from the potential participants. So I expect, or we do expect that, that should form a, some sort of unification of the capacity to communicate about the research to the potential participants. And the rest of it will be individual differences which really, not really, you really can’t do much about this.* [Clinician and academic]

A prominent *ethics-related* perspective covered how CE staff approached communities and that this did not always reflect that staff held a holistic view of participants and the various individual, interpersonal, socio-cultural, and environmental factors they are affected by. It appeared that not having an informed and comprehensive understanding of the communities, the members, the existing socio-cultural landscape, languages, those positioned as leaders, and the rules of engagement, hampers CE and the success of the clinical trial. Reflecting on effective approaches for conducting research with communities, a CE worker noted the following:

*There is quite a lot in the approach that, you know, that adds to, [that] one could find difficult for [the] researcher to engage communities. I mean with this whole elitist image that you got as a researcher, you’ve got to play that down significantly…instil humbleness. Bring yourself down to that level. Find a person who can lead the engagement if you are struggling to get to a comfortable place where you can be at the same level where people feel like you are engaging honestly. Find somebody in your team who can do that and let them lead the engagement–let them be the face of the engagement, let them be the person who is in front speaking or facilitating the conversation. Because at the end of the day what you want is an honest conversation with the participant where the information is seen as credible. And you don’t want any barrier that makes it hard for the participant to give you good information, and also to come back because they feel same and comfortable enough to be part of the study. So, I found that getting somebody who can engage at a, in a way that people in the community can say you know what, I feel safe here–I know I can talk to these people, or you know, these people are actually honest–let’s be part of this or let me be part of this. And you will be shocked to see how these guys, they talk. Like if there was a study and it was led by a person and this person or this team was very difficult in the sense that they created bad relationships and they are from your University, that is, ah, by the time you go into that community people are still going to remember and they are talking to each other and they will be telling each other no, don’t be part of it.* [CE worker]

Reflecting on their previous engagements with local communities, the CE worker noted that “…where I have gone into the community at a very top-down approach, even when I know what the issues are, those wards, those relationships didn’t really last and I didn’t get the kind of support I needed and had to do more work to try and rebuild those relationships—that’s a lot of money and time wasted. So ja [yes], there’s definitely a way of approaching these things that actually makes a difference”. These views were prominent in other respondents’ narratives as well, when they discussed the need to approach community leaders in a way that showcases an understanding of the rules of engagement in communities and the structures wherein the communities function. Pertaining to the latter, a professor held the following view.

*Another thing is how we, the attitudes that we go to the community with, how we want them to see us. How you want to address them, how you want to define the relationship. And that is the important [thing], because it will determine what they demand from you. You go with an attitude of I am the researcher, I have the money, then they are all going to look towards you for the money, but if you go with you are the researcher here, you are the one who would give the answers to your problems and it’s your problem, or what you perceive to be a challenge. And that is the first thing actually–they must see it as a problem–they must see whatever it is that you are going to, you want to engage in, they must see it as theirs. It’s something they need to solve for themselves. So those two key things are important, relationship with the partners, with the academics and … And another thing is they need a long-term plan on what the outcome of this is going to be. How is it going to benefit them in the long run–they need to have that.* [Clinician and academic]

*These are conservative traditional communities*. *There are certain things you cannot say when you are in stakeholder engagement with churches or traditional leaders*, *that you can say in another forum*. *And be mindful*, *if you’re going to raise a certain topic–let’s say around*, *I don’t know*, *use of condoms or abortion rights–you’re going to talk about those topics*, *you need to understand how you need to introduce it so that if you want church leaders in the room*, *they can also have something to say about it without feeling*, *you know*, *as*, *you know*, *everybody is sort of agreeing with an agenda that they don’t have a clue about*. *So they are kind of democratizing spaces*, *making it really equal for everybody to have a voice*, *and so you position yourself at some point as a person who has got the power*, *you know*, *chairing the meeting*, *and then you move away from that chairperson who just really wants an agenda to emerge from the room*. *You know*, *I don’t know how else to say it*, *you just need to be so mindful and tactful*. *That’s the word*, *you just need to be tactful in how you approach this discussion*. [CE worker]

Multiple CE stakeholders associated a holistic view of communities, their structure and *make-up*, as key considerations to ethical conduct in research, as it lends to the idea that a community is a complex system. It appeared that respondents gave thought to strategies for engagement and provided examples of guidelines that could be effective. This included a participant instructing that: “…you’ve got to position yourself as empathetic with their (i.e. community members’) issues. And you’ve got to be able to show that you really see why they care about what they care about, why it is a big problem, and explore that with them, so make time for that in the relationship-building plays of the stakeholder engagement”. The latter viewpoints introduce the next theme which covers the opportunities for improved CE practices.

#### Theme 4: Opportunities for improved CE practices

Two sub-themes were prominent when considering strategies for strengthening CE in clinical trial research. These are a) develop and implement standardised procedures for CE and b) considerations when approaching communities.

*A*. *Develop and implement standardised procedures for CE*. A pervasive narrative among respondents was that developing and implementing standardised research and ethics procedures is key to effective CE, and that training for CT staff should foster this. The proposed procedures mostly pertained to how fieldwork, recruitment and general CE practices were approached. A clinical researcher described standardised procedures as providing “a level playing ground” whereby the room for error can be reduced since everyone would provide the same information to potential participants. Related to this, a CE manager proposed that a forum for discussing collaboration, CE and challenges would strengthen CE practices in CT, stressing the need for this type of forum as an opportunity for ideas-exchange and support.

*So, but on a level playing ground, everybody receives the same information during the training session and what is expected of each person is also explained during these sessions. So basically there is base line expected participation.* [Clinician and academic]

In relation to standardisation, respondents emphasised that adherence to ethical practice prior to, during, and after the study was key to improved CE in clinical trial research. A CE officer described that for successful CE to be fostered, researchers should not “forget” to incorporate CE in grant applications. This was a common view expressed. It was noticeable that there was a recognised gap (and opportunity) for integrating CE procedures within the early stages of the research process, instead of CE being viewed as an addendum. To reiterate sentiments expressed earlier, a CE worker noted the following:

*The CE team gets involved too late, where we didn’t have enough time to make sure that there is enough budget, to make sure that like, early, like early enough engagement is happening within those communities where a protocol is written and only after that sites are selected where it’s kind of, ja, it makes it very hard.* [CE worker]

While these early-stage considerations were stated, respondents also discussed the importance of research processes post-trial, for example, when describing the importance of data sharing involving trial participants.

*My current focus [is] ensuring that investigators look at the issues around access post-trials, not just to participants, but the communities that pro-, contributed towards this finding.* [CE manager]

Linguistic considerations were also mentioned as essential to standardised methods for CE. These considerations included ensuring that research documents, especially consent forms and initial recruitment conversations, are accessible to the population in terms of their literacy levels and their preferred languages. In this regard, it was emphasised that using a translator for these processes would be essential in ensuring that consent is sought in a standardised and ethical way. These perspectives are shared by CE teams and researchers. Below are the views of a community manager.

*A science must be able to be translated. It must translate a science to…a lay person…it’s one of the things that I see, it is like a gap, because a person who is the…a person who is, at the ground, might not understand some of the terms, so it is always important to ensure that the language is understood, is shared, and the reasons for doing that particular study. I think those are the most critical aspects in conducting research.* [CE manager]

Respondents indicated that there is a gap in knowledge amongst different research staff, especially pertaining to understanding research concepts, commenting that “…it can be overwhelming and we’re talking about studies and it’s numbers and all these randomisation, informed consent and placebo”. Moreover, contributing to this call for research-literacy, a CE worker stated that their liaison/engagement staff need to be empowered extensively through clinical research training, which would enable them to effectively collaborate with stakeholders and community representatives.

*We want to like enable them to also provide GPP training to their CABS, to different stakeholders, we want them to be able to provide research literacy training on specific topics when needed. And then we’re also doing the, this vacillate library [inaudible 25.38]. So the vacillate library is something that we’re developing at the moment and it’s a very comprehensive set of like vaccine literacy topics. So it covers all of the diseases now that we are doing like the history of TB and why vaccine is needed and how vaccine studies work, and new approaches and different ways of doing clinical trials. So that’s going to be a comprehensive list of like a whole work package module library with different materials.* [CE worker]

*B*. *Considerations for approaching communities*. Respondents indicated that there are certain guidelines for interacting within community spaces and with different stakeholders (including community representatives and current/future study-participants). CE staff felt that these processes should be respectful, ethical, and echoed that communities are unique and complex systems. There are two distinct perspectives evident in this topic, namely a) engaging with trial participants as people within and outside of the research study, and b) acknowledging community contexts as complex, unique systems.

***Engaging with participants as people within and outside of the research study*.** A dominant shared-perspective among respondents was the need to develop a holistic understanding of communities and that *participants are people* that are diverse, have varied needs, and who are complex in terms of the internal, interpersonal, socio-economic, cultural, contextual, and historical dimensions that affect them. Adopting this lens was seen as beneficial to facilitating successful CE and positioned as a strategy that can be adopted by CT staff.

*Researcher: What really enables effective or successful CE for those stakeholders?*

*Respondent*: *In my experience it really is*, *you have got to position yourself in two ways*. *One*, *you’ve got to position yourself as empathetic with their issues*. *And you’ve got to be able to show that you really see why they care about what they care about*, *why it is a big problem*, *and explore that with them*, *so make time for that in the relationship-building plays of the stakeholder engagement*. *And secondly*, *you’ve got to position yourself as supporting what they are doing already*. *So not trying to duplicate things and create another layer so that you don’t look competitive*. *So*, *strange enough*, *people are competing for these spaces and that’s just sort of human nature in [these] kind of spaces*. *People hold those spaces very dear*, *and if somebody else comes and they look [as if] they are taking away from their impact or their relevance*, *then they don’t have a very easy relationship with you*. [CE worker]

Further ideas for future successful CE, was ensuring that staff engage in a way that underscores their understanding of the significant socio-economic challenges that participants face and that they respect participants’ right to have precise information about benefits and limitations in the study. Talking about this, one respondent shared the following thoughts:

*People have become extremely sensitive to what they can benefit from being part of a research study and so on. So, the approach that you need to use is one that shows that you understand this, you know that people are thinking this, and you are clear about being very communicative around this very matter. Don’t run away from it, deal with it. And also, be welcoming of [the] kind of conflict that arises because of this perception of who you are. And understand that it’s also a perception that could have been formed before you came, so chances are you are not even responsible for this perception that people have of you. And be willing to spend time with people who worry about this, this issue, especially if they are like power brokers in the community and they are the ones who give you access to other people in the community that you want to talk to, you want to engage in your study. So, you spend time with that, and that’s the approach that at least I have, which you know, give CE the kind of time that it needs.* [CE worker]

Several other respondents mentioned the latter notion of *transparency about the study details* and that promises about financial benefits should not be made if it is not true or confirmed (especially in the context of poverty and the need for funds to cover basic needs). The narrative captured below points to proposed ways to address this and avoid false-hope about benefits.

*The second thing is make promises you can keep. Do not promise people that they are going to be, I don’t know, funded if they’re [not] or even say things like there is potential for other funding that could come your way if you are part of our process. And rather change the discussion to something like if you’ve got a guide proposal or you have a proposal you are working, we are more than willing to find a system we can and strengthen that proposal based on our experience. And then people could have knowledge that you can only help so far. Oftentimes we are seen as having a bucket load of money and can make things happen very quickly, and that’s really not the case. So, an approach that kind of shows that you are sensitive, empathetic for their struggles, their issues that they are raising, issues that you see relevance in, you know, you think they are relevant. An approach that shows that you are going to be honest from the start about what you can and cannot do.* [CE worker]

Another guideline informing how communities should be approached is related to information-sharing. In this case, a CE and research manager’s priorities in clinical trials was rooted in knowledge translation as they discussed the pertinence of equity in research and post-trial access to information for participants and the broader community.

*The second part is just ensuring that we understand, starting with myself, the concept of data sharing, data ownership, what is the data agreement…on those issues. So, moving more towards the issues around equity in…the research frame.* [CE manager]

These sentiments are also associated with the notion that research organisations must consider communities outside the borders of the research, particularly in relation to their health-needs. There was a perceived need that CT staff should “continue talking to them [participants] about maybe HIV, depending on the disease indication”, possibly to address concerns such as “…why should people participate in these things if honestly we are extracting something and not giving anything back”. In adding to which strategies might be helpful for CE, a director of CE who previously worked as a fieldworker summarised how clinical trial staff can “give back” to the community in a way that aligns to their right to receiving health information and post-trial support:

*When the study ends at the phase of data analysis, maybe some community people need some feedback, maybe they want some kind of engagement, really. Continued talking to them about maybe HIV, depending on the disease indication. And you are not there. People have questions to ask. They are not seeing you anymore. Yet, initially you built a relationship where you mentioned to them that you will be able to provide them health education, you will be available to answer their health concerns. something like that. So, the pieces are really the breakdown of information, but also the breaking of the communication and the budget. For me I think that’s the highlights that I can mention.* [Head of CE]

On the same topic, a respondent with extensive experience in research and CE roles discussed how the end of a trial also signals the end of quality health care and support that people depend on, and which prompt some participants to want to remain part of a study.

*You start with…the paediatric trial and you…work with a child, and the child kind of grows up, you know, during the study with you, and then co-, you know, they … they get older and they don’t want to leave, and a lot of participants don’t want to leave the research, like one … like a research unit like ours, because they feel they get a better quality of healthcare … or what do you call it? Like…you know, someone looking out for them.* [Researcher]

***Acknowledging community contexts as complex*, *unique systems*.** Respondents suggested that clinical trial staff must have an awareness and understanding that communities operate within an existing structure with its own rules of engagement and the role of the researcher is not to disrupt existing processes, but to slot into these structures (using ethical procedures).

*But also, we need to better understand that particular community and then also then develop a particular approach… to say then how are we going to work with this particular community. There is no one-size-fit-all when it comes to community entry. But also, part of the CE also is to further educate them about the studies that we are…doing currently and probably past studies.* [Head of CE]

Pertaining to this, one respondent declared that they had a “duty” to integrate community consultation into early stages of engagement processes and also alluded to how this could have a positive impact on the research process (i.e. via identifying gatekeepers early):

*If we have a particular study, then my duty is to go first in the community and have a meeting with the different role players, different people, different gate keepers, [to] identify gate keepers, just to basically, to do the research, because each and every community entry is different from each and every community. Some of the community we have influence, some of the community we have no influence.* [Head of CE]

A lack of insight into the role of traditional and cultural structures within certain contexts was said to significantly affect how CE is approached and positioned as an opportunity for improved CE practices. It was equally emphasised that, while extensive *homework* must be done about the community, the individual approach and identity of the CT staff members seemed to matter as well. This included how they have aligned themselves with a political organisation, their culture, ethnicity, race, language, gender, their home-area, as well as how their attitude is perceived by community leaders (notably, “first impressions matter”). One respondent declared that “…as a researcher you’ve got to be able to know, so that you can, you know, position or get somebody else, position yourself or get somebody else to help you along…”. This seems to allude to the importance of having knowledge of the community and its members that is necessary to aid the researcher with deciding how to approach CE effectively. Related to this, a clinical researcher and director of CE respectively discussed specific practices that must be adopted to facilitate contextually and culturally-informed CE:

*First impressions matter a lot. So, you have to really prepare for that very first meeting. You need to do some background checks, you need to really resolve who is the most important, or who are the most important people, because apparently in communities there could be factions. So, if you get to talk [to] the leader…that is seen like a faction head, it precludes you from being able to meet with all the others, or the opposing parties. So, you need to not inadvertently play into a rivalry within the community so that’s the need for a background check. Randomly speak with community residents to give you an idea, a balanced information as regards who are the focal people that need to be seen. Then when that is done, you make formal requests to engage with them, clearly stating the business for which you want to engage with them, and in many situations you have to clearly dissociate yourself from government or political affiliations or whatever affiliations create a divide, so as not to inadvertently shoot yourself in the foot.* [Clinician and academic]

*You need somebody*, *if you want to relate with the cultural leader*, *the primary ones*, *one depending on which culture you are*. *For example*, *if you are in South Africa and*, *and you want to get in a community that has a lot of*, *say there is Zulu population*, *you need to find a Zulu representative*. *And the Zulu king representative in the community*. *First help that one understand what you are talking about and then they will probably struct… they have systems and meetings and committees*, *sometimes actually they have health committees that take care of discussing health related issues*, *they have a parliament*, *they have leadership structures*, *so work with that leadership and*, *and educate them*. *Sometimes you can even have the opportunity to educate the King about the new intervention*, *depending on how the engagement needs to*, *which approach it needs to take*. [Head of CE]

All respondents had research-experience within African contexts, and many discussions included the view that traditional/community leaders’ practices and expectations must be considered extensively since as they are gatekeepers to community members. This being said, it was noted that the approach with each community representative itself cannot be duplicated, further emphasising the importance of learning about the community before a study commences.

*If, when you are speaking to the elders, how then do you conduct yourself when you’re speaking to Nkosi [refers to traditional leader in the community, also known as a chief], how then are you conducting yourself when you are speaking to a school principal? It is a different setting altogether. You find that you need to change your approach in one meeting…maybe 4 to 5 different times, because the way how you speak to Nkosi is different how you speak to [the] principal. The way you speak to a principal is different. It should be also different because the level of understanding is also different when it comes to ordinary community members. The way how you explain yourself and you explain things, it need[s] to be simple as possible, in a layman’s language. Not even layman, but in a community’s language; things that we call on a day-to-day. But then there are also other hurdles that we also experience at the community level. You’ll find that the language development, it’s way behind than science. Science is developing very fast. There are words that we do not have in our vocabulary.* [Head of CE]

In summary, the respondents that were interviewed for the study discussed various challenges they faced in their personal roles and described perceived challenges within CE from the perspective of the community. While a range of threats to effective CE was argued for and stressed, respondents also suggested strategies that may aid successful CE if it is implemented by CT staff when working with people in complex community-systems.

## Discussion

This qualitative study aimed to investigate CE in infectious disease clinical trials in SSA from the perspectives of CE stakeholders. Findings showed that these stakeholders often viewed communities as *abandoned* research entities that are only important during the recruitment phase. On the topic of importance, CE teams, including fieldworkers and liaison officers, felt that investigators did not regard their roles as important for the research process and consequently felt undervalued and ’left out’ of important conversations pertaining to CE. Critical perspectives about CE revealed a range of ethical concerns and gaps. However, stakeholders reflected significantly on potential avenues for improved CE.

Pertaining to theme one, it was evident that teams working primarily with communities, like fieldworkers and liaison officers, felt that clinical trial staff, like investigators, only cared about community members at the beginning of a study for the purposes of enrolment. They noted that once research-teams have enrolled the required sample, *communities were abruptly abandoned*, no longer communicated with, and did not receive beneficial health/educational-information following their participation in a trial. This lack of end-of-trial health information provided to participants was further problematised since academic outputs remained a priority and therefore the research community was provided with end-of-trial information, but research participants or communities were not. Sentiments therefore spoke to whether participants are valued, whether their health is a priority, or whether they were merely numbers for a clinical trial. These findings are consistent with previous research emphasising the need for researchers to foster long-lasting relationships with communities; relationships that are not comprised of once-off endeavours, but that carries through to the end of a trial [11]. The latter study was implemented in South Africa and entailed qualitative interviews with CAB members and CT staff, and it was apparent that CE stakeholders called for taking responsibility for nurturing a relationship with communities, but also to responsibly and diligently share information with the communities about relevant health issues and the research process.

In terms of theme 2, and expanding on the perspectives of key bi-directional, supportive, and nurtured relationships with communities in clinical trial research [11], CE teams involved in this study had concerns about the *disconnected relationship between investigators and CE teams*. We found that CE teams were dissatisfied about numerous things, including being excluded from key research processes at early stages and having a feeling of being valued only until the required sample is enrolled. CE teams seemed to allude to their roles and CE being perceived as an "advertisement" by other team members and that recruitment was viewed as the extent of CE’s value. This is in contrast to how CE teams conceptualise their roles. A community liaison officer (who was not part of this study), for example, emphasises that their role is not about approaching people as "numbers" for recruitment purposes, but about viewing people in a more holistic sense and being sensitive to their health-needs and their role in trying to aid them (i.e. referral/health information) [22]. CE teams’ perspectives of exclusion from research processes, their roles being an ‘after thought’ and feeling that they are not respected, conflicts with the fundamental ingredients of functioning clinical trial teams, namely holding “mutual respect for each team member’s role” [23], as an essential pathway to the successful clinical trial implementation.

The third theme of the study spoke to the ethical considerations and areas for improvement from the perspectives of various CE stakeholders. A core ethical consideration among stakeholders was concerns related to questionable research literacy for both CT staff and participants. Problematic research literacy manifested in various ways, including:

1. A lack of standardised procedures were used by various staff members when communicating essential study information to communities and participants, and
2. Some staff do not understand key research concepts that they share with potential participants.

It was apparent that CE teams were sometimes expected to present information to advisory boards and communities as part of enrolment, but that they were not adequately informed about the study (and excluded from key meetings), and in some cases CT staff did not sufficiently grasp the research concepts or study-information they discussed with communities and potential participants. This begs the question whether informed consent is underpinned by ethical procedures. *is it ethical for people to consent to a clinical trial that they were not sufficiently informed about*? The problematic aspects of a lack of research literacy is corroborated by other multi-country research. For example, Newman and associates (2015) conducted a case study involving a diverse group of clinical trial stakeholders. The findings emphasised that research literacy was critical to trial implementation, but that several factors impeded the CT staff’s ability to effectively communicate the nature and impact of the trial with potential participants including a lack of research knowledge. In our study CT staff corroborated this, noting that “I remember when I started in research…it was so difficult for me, you know, research has their own language”. In agreement, a participant in the Newman study pointed to inadequate trial information being shared with them before they participated in a study, saying that they “at least” would like to be informed about what a vaccine is before participating, highlighting a gap in how trial-information is shared as reported in other studies [24] and in the present study.

It is notable that community members are unique and complex in terms of the individual, interpersonal, contextual, cultural, and historical factors they are affected by. In this study, CE stakeholders significantly problematised how CT staff approached and attempted to build relationships with communities, with the underlying message being that communities are not always treated with respect or consideration for their existing leadership structure, their diversity, complex socio-economic and health needs. This includes a need for health information and the importance of equitable connections. Examples of the types of issues not approached with sensitivity and due consideration were 1) not accounting for traditional leadership structures and what this means for gaining access to community members’ perspectives, 2) not providing health or post-trial information and therefore “not giving anything back” to communities and participants, and 3) not accounting for socio-economic benefits that may inform participation for people affected by poverty and ensuring that benefits (or a lack thereof) are made clear.

A study by Kaehler and colleagues (2021) echo most of these sentiments. The latter study involved qualitative interviews with policymakers and researchers in the Greater Mekong sub-region in Asia. Similar to our findings, the researchers highlighted the need for considering local “authorities”, which would include community leadership structures, as part of CE, and being mindful of possible benefits that can be provided to participants that considers their context (i.e. providing health services to participants living in remote areas).

The fourth theme of the study refers to *opportunities for improved CE* and captures perspectives about ‘what’ could address the ethical concerns (and other factors) discussed earlier. One sub-theme speaks to developing and implementing standardised procedures for CE. This includes 1) involving CE staff in core research meetings throughout the research process, 2) ensuring that CE procedures and activities are accounted for before the trial commences, 3) involving communities in research processes, and 4) ensuring that training addresses some of the needs for skills-development that CT staff have. Pertaining to the latter point, there was a need for training that fosters the use of rigorous and standardised CE procedures among all CT staff. The need for improved research skills and research literacy was clearly identified as an aspect that training should address since there were concerns about CT staff being able to communicate research concepts to communities/potential participants accurately and in an accessible way that avoids only using research jargon. The importance of research literacy in CE is equally emphasised by other authors studying CE and ethical practices in SSA and who also recognised the importance of fostering research literacy amongst CT/CE stakeholders [25].

A key message emerging in this study is that CT staff should carefully consider and revise how they approach communities as part of research studies. In this regard there is a notion that community members might be part of a trial, but they are unique individuals with their own intrapersonal and social characteristics and are embedded in a multi-facetted context with its own historical, cultural, and social dimensions. CE stakeholders stressed that communities must be approached in a way that recognises these complexities and that formative research about relevant communities is crucial in clinical trial research. These perspectives are shared by others, noting that formative research can inform CT staff about key historical, social, and political factors that can shape community participation in trials and that “community engagement programmes (outlined in a developed community engagement plan) should be grounded in the social practices and norms of the community thereby allowing for prompt identification and response to community specific issues that may otherwise impede or delay the engagement process” [4]. Consequently, integrating formative research about relevant communities as a requirement for clinical trials could help foster an awareness of key variables affecting how communities should be approached, and how connections and collaboration can be sustained.

As part of gaining a holistic understanding of communities, CE stakeholders emphasised that respect towards communities and existing leadership structures were important, especially since traditional structures in SSA are common. The literature speaks to this as well, clarifying the value of building trustful and supportive relationships with community leaders [13] which might positively influence access to communities. In contrast, CE stakeholders called caution to the opposite effect, whereby failure to account for the influence of community leaders could lead to communities becoming inaccessible. As part of efforts to improve CE, it appears that training programmes could include strategies for conducting standardised formative research based on the communities wherein clinical trials would be conducted. This could help researchers “create a historical and socio-cultural map of the relevant communities” [4] which can help inform their engagements with communities and participants.

## Conclusion

This study identifies the multi-level factors affecting CE in clinical trials in SSA from the perspectives of various CE stakeholders. Pertaining to strategies for improved CE practices, it may be beneficial to revise and/or enhance current training programmes for CT staff. A dominant message in this study is that CE procedures still require standardisation among CT staff. This is to ensure that all CT staff provide standardised study-information to communities, which should be linguistically appropriate and accessible to people who are not familiar with research concepts. Pertaining to clarifying research concepts, it was notable that training should foster research literacy among CT staff as well, which could enhance their ability to communicate essential study-information to community members. It is apparent that training processes should also include efforts to facilitate an enhanced understanding of the multi-level individual and environmental factors affecting communities and how this influences their engagement in clinical trial research. This can be done through formative research about communities.

While a strength of this study is its involvement of CE stakeholders from diverse backgrounds and contexts in SSA, a notable limitation was the void of former/current CT participants and them sharing their views on CE. We have learned lessons about how we approached these communities for their participation and note that, while we used various methods of engagement, none yielded participation from trial participants. This points to the need for revising CE and recruitment practices in general health research. Perhaps future research can explore creative, culturally and contextually informed strategies for reaching these populations that extend further than recruitment via CT and research stakeholders, organisations, advocacy groups and social media. It would be imperative to understand the complexities of CE from the perspectives of those directly affected by it and, therefore it is a non-negotiable for communities and trial participants to be included in the conversation about CE.

## References

[1] World Health Organization. HIV/AIDS fact sheet. 2023. Available from: https://who.int

[2] Mbuya AW, Mboya IB, Semvua HH, Mamuya SH, Msuya SE. Prevalence and factors associated with tuberculosis among the mining communities in Mererani, Tanzania. PLoS One. 2023;18(3):e0280396. Available from: doi: 10.1371/journal.pone.0280396 36920939 PMC10016659

[3] Cresswell FV, Kasibante J, Martyn EM, Tugume L, Stead G, Ssembambulidde K, et al. A journey of hope: giving research participants a voice to share their experiences and improve community engagement around advanced HIV disease in Uganda. AAS Open Research. 2020;3 (33). Available from: 10.12688/aasopenres.13104.2 PMC7682503 33274313

[4] Folayan MO, Durueke F, Gofwen W, Godo-Odemijie G, Okonkwo C, Nanmak B, et al. Community stakeholder engagement during a vaccine demonstration project in Nigeria: lessons on implementation of the good participatory practice guidelines. Pan African Medical Journal. 2019;34. Available from: doi: 10.11604/pamj.2019.34.179.18458 32153719 PMC7046105

[5] Simwinga M, Bond V, Makola N, Hoddinott G, Belemu S, White R, et al. Implementing community engagement for combination prevention: lessons learnt from the first year of the HPTN 071 (PopART) community-randomized study. Current HIV/AIDS reports. 2016;13(4):194–201. Available from: doi: 10.1007/s11904-016-0322-z 27405816

[6] Saluzzo F, Espinosa-Pereiro J, Dressler S, Tàvora Dos Santos Filho E, Seidel S, Gonzalez Moreno J, et al. Community engagement in tuberculosis research: the EU-Patient-cEntric clinicAl tRial pLatforms (EU-PEARL) experience. International Journal of Infectious Diseases. 2023;130:S20–S24. Available from: doi: 10.1016/j.ijid.2023.03.008 36906120

[7] Dada S, McKay G, Mateus A, Lees S. Lessons learned from engaging communities for Ebola vaccine trials in Sierra Leone: reciprocity, relatability, relationships and respect (the four R’s). BMC Public Health. 2019;19(1). Available from: doi: 10.1186/s12889-019-7978-4 31829223 PMC6907283

[8] Bain LE, Akondeng C, Njamnshi WY, Mandi HE, Amu H, Njamnshi AK. Community engagement in research in sub-Saharan Africa: current practices, barriers, facilitators, ethical considerations and the role of gender—a systematic review. Pan African Medical Journal. 2022;43(152). PMC9922083 doi: 10.11604/pamj.2022.43.152.36861 36785694 PMC9922083

[9] Marsh V, Kamuya D, Rowa Y, Gikonyo C, Molyneux S. Beginning community engagement at a busy biomedical research programme: experiences from the KEMRI CGMRC-Wellcome Trust Research Programme, Kilifi, Kenya. Social Science Medicine. 2008;67(5):721–33. Available from: doi: 10.1016/j.socscimed.2008.02.007 18375028 PMC2682178

[10] Akondeng C, Njamnshi WY, Mandi HE, Agbor VN, Bain LE, Njamnshi AK. Community engagement in research in sub-Saharan Africa: approaches, barriers, facilitators, ethical considerations and the role of gender–a systematic review protocol. BMJ Open. 2022;12(5):e057922. Available from: doi: 10.1136/bmjopen-2021-057922 35545398 PMC9096545

[11] Dietrich JJ, Munoz J, Tshabalala G, Makhale LM, Hornschuh S, Rentas F, et al. A qualitative study of stakeholder and researcher perspectives of community engagement practices for HIV vaccine clinical trials in South Africa. Journal of Community Psychology. 2023;51(3):998–1015. Available from: doi: 10.1002/jcop.22951 36342974 PMC10613584

[12] Kaehler N, Adhikari B, Cheah PY, von Seidlein L, Day NPJ, Dondorp AM, et al. Community engagement for malaria elimination in the Greater Mekong Sub-region: a qualitative study among malaria researchers and policymakers. Research square pre-print. 2021. 10.1186/s12936-022-04069-x PMC8845385 35164770

[13] Barugahare J, Kass NE. Managing community engagement in research in Uganda: insights from practices in HIV/AIDS research. BMC Medical Ethics. 2022;23(1). Available from: doi: 10.1186/s12910-022-00797-6 35701777 PMC9199168

[14] Downe S, Finlayson K, Tunçalp Ö, Gülmezoglu AM. Provision and uptake of routine antenatal services: a qualitative evidence synthesis. Cochrane Library. 2019;6. Available from: doi: 10.1002/14651858.CD012392.pub2 31194903 PMC6564082

[15] UNAIDS. Good participatory practice: guidelines for biomedical HIV prevention trials. 2011. Available from: https://unaids/org

[16] UNAIDS. Ethical considerations in biomedical HIV prevention trials [additional guidance point added in 2012]. 2012. Available from: https://unaids.org

[17] UNAIDS. Global HIV statistics. 2023. Available from: https://unaids.org

[18] Braun V, Clarke V. Using thematic analysis in psychology. Qualitative Research in Psychology. 2006;3(2):77–101. Available from: 10.1191/1478088706qp063oa

[19] Department of Health. Ethics in health research: principles, processes and structures. South African Government, Department of Health. 2015. Available from: https://knowledgehub.health.gov.za/elibrary/ethics-health-research-principles-processes-and-structures

[20] World Medical Association. World Medical Association Declaration of Helsinki ethical principles for medical research involving human subjects. JAMA: Journal of the American Medical Association. 2013;310 (20): 2191–2194. doi: 10.1001/jama.2013.281053 24141714

[21] HIV Prevention Trials Network (HPTN). HPTN Community working group. 2024. Available from: https://www.hptn.org/community/community-engagement-program/CWG

[22] IAVI VOICES. Community perspectives on clinical research. 2020. Available from: https://www.iavi.org/our-work/advocacy-community-engagement/

[23] Baer AR, Zon R, Devine S, Lyss AP. The clinical research team. Journal of Oncology Practice. 2011;7(3):188–92. Available from: doi: 10.1200/JOP.2011.000276 21886502 PMC3092661

[24] Newman PA, Rubincam C, Slack C, Essack Z, Chakrapani V, Chuang D-M, et al. Towards a science of community stakeholder engagement in biomedical HIV prevention trials: an embedded four-country case study. PLoS One. 2015;10(8): e0135937. Available from: doi: 10.1371/journal.pone.0135937 26295159 PMC4546590

[25] Folayan MO, Peterson K, Haire B, Brown B, Audu K, Makanjuola O, et al. Debating ethics in HIV research: gaps between policy and practice in Nigeria. Developing World Bioethics. 2015;15(3):214–25. Available from: doi: 10.1111/dewb.12064 24975983 PMC6863742

